# Optimization of Genomic Selection to Improve Disease Resistance in Two Marine Fishes, the European Sea Bass (*Dicentrarchus labrax*) and the Gilthead Sea Bream (*Sparus aurata*)

**DOI:** 10.3389/fgene.2021.665920

**Published:** 2021-07-14

**Authors:** Ronan Griot, François Allal, Florence Phocas, Sophie Brard-Fudulea, Romain Morvezen, Pierrick Haffray, Yoannah François, Thierry Morin, Anastasia Bestin, Jean-Sébastien Bruant, Sophie Cariou, Bruno Peyrou, Joseph Brunier, Marc Vandeputte

**Affiliations:** ^1^SYSAAF, Station LPGP/INRAE, Campus de Beaulieu, Rennes, France; ^2^Université Paris-Saclay, INRAE, AgroParisTech, GABI, Jouy-en-Josas, France; ^3^MARBEC, Univ. Montpellier, Ifremer, CNRS, IRD, Palavas-les-Flots, France; ^4^ANSES, Ploufragan-Plouzané-Niort Laboratory, Viral Fish Diseases Unit, National Reference Laboratory for Regulated Fish Diseases, Technopôle Brest-Iroise, Plouzané, France; ^5^Ferme Marine du Douhet, La Brée Les Bains, France; ^6^Ecloserie Marine de Gravelines-Ichtus, Gravelines, France

**Keywords:** genomic selection, *dicentrarchus labrax*, *Sparus aurata*, disease resistance, aquaculture

## Abstract

Disease outbreaks are a major threat to the aquaculture industry, and can be controlled by selective breeding. With the development of high-throughput genotyping technologies, genomic selection may become accessible even in minor species. Training population size and marker density are among the main drivers of the prediction accuracy, which both have a high impact on the cost of genomic selection. In this study, we assessed the impact of training population size as well as marker density on the prediction accuracy of disease resistance traits in European sea bass (*Dicentrarchus labrax*) and gilthead sea bream (*Sparus aurata*). We performed a challenge to nervous necrosis virus (NNV) in two sea bass cohorts, a challenge to *Vibrio harveyi* in one sea bass cohort and a challenge to *Photobacterium damselae* subsp. *piscicida* in one sea bream cohort. Challenged individuals were genotyped on 57K–60K SNP chips. Markers were sampled to design virtual SNP chips of 1K, 3K, 6K, and 10K markers. Similarly, challenged individuals were randomly sampled to vary training population size from 50 to 800 individuals. The accuracy of genomic-based (GBLUP model) and pedigree-based estimated breeding values (EBV) (PBLUP model) was computed for each training population size using Monte-Carlo cross-validation. Genomic-based breeding values were also computed using the virtual chips to study the effect of marker density. For resistance to Viral Nervous Necrosis (VNN), as one major QTL was detected, the opportunity of marker-assisted selection was investigated by adding a QTL effect in both genomic and pedigree prediction models. As training population size increased, accuracy increased to reach values in range of 0.51–0.65 for full density chips. The accuracy could still increase with more individuals in the training population as the accuracy plateau was not reached. When using only the 6K density chip, accuracy reached at least 90% of that obtained with the full density chip. Adding the QTL effect increased the accuracy of the PBLUP model to values higher than the GBLUP model without the QTL effect. This work sets a framework for the practical implementation of genomic selection to improve the resistance to major diseases in European sea bass and gilthead sea bream.

## Introduction

Viral and bacterial infectious diseases are a major threat to the development of aquaculture production (Gjedrem, 2015). In the Mediterranean Sea, fish culture is mainly focused on European sea bass (*Dicentrarchus labrax*) and gilthead sea bream (*Sparus aurata*) with a production of 157,000 and 160,000 tons, respectively in 2016 (FEAP, 2017). Viral Nervous Necrosis (VNN) caused by the nervous necrosis virus (NNV), vibriosis caused by *Vibrio harveyi* and pasteurellosis due to *Photobacterium damselae* subsp. *piscicida* are considered the most impacting diseases for Mediterranean aquaculture (Vendramin et al., 2016). They cause high mortality in aquaculture farms and both prophylaxis and therapeutics remain insufficient to control outbreaks.

Selective breeding to improve the genetic resistance to those pathogens is a promising approach to prevent outbreaks. It has been successfully applied in many aquaculture species and led to a genetic gain of 12.5% in disease resistance per generation on average over a number of host–pathogen pairs (Gjedrem and Robinson, 2014). To select for genetically resistant individuals, enough genetic variability must exist within the species. Moderate to high heritability (0.24–0.43) has been reported for resistance to VNN in European sea bass (Doan et al., 2017; Palaiokostas et al., 2018; Griot et al., 2021) and for resistance to pasteurellosis in gilthead sea bream (from 0.22 to 0.32) (Palaiokostas et al., 2016; Aslam et al., 2018), thus presenting opportunity for genetic improvement.

Genomic selection is a tool that can increase the efficiency of selective breeding. Classical pedigree-based selective breeding derives genetic relationships between the individuals from pedigree records and combines them with phenotypes to estimate breeding values. Genomic selection uses genomic markers spread along the genome to estimate those genetic relationships more accurately, leading to better estimates of breeding values (Meuwissen et al., 2001). For most of the traits studied in aquaculture, genomic selection indeed outperforms pedigree-based selection and increases the genetic gain per generation (Nielsen et al., 2011; Zenger et al., 2019).

Genomic selection is particularly interesting to improve disease resistance because phenotypes are collected on related individuals instead of the selection candidates themselves, in order to avoid contacts between the pathogen and the selection candidates. The genomic breeding values of the selection candidates are predicted based on the phenotypes recorded on challenged relatives and the markers genotyped on both challenged individuals and selection candidates (see review of Ødegård et al., 2011). When the phenotype is collected on relatives and not on the candidates, pedigree-based selection only accounts for between-family genetic variance, but genomic selection can account for both between and within-family genetic variances and thus, allows to rank the individuals within their family, which increases the precision of estimated breeding values (EBV). In the European sea bass, genomic selection has been shown to increase prediction accuracy by 13% to improve VNN resistance compared to pedigree selection (Palaiokostas et al., 2018). In the gilthead seabream, genomic selection was shown to outperform pedigree selection to improve pasteurellosis resistance, improving prediction accuracy by 27–53% (Palaiokostas et al., 2016). Those two studies provide appealing results, but only compare the efficiency of genomic and pedigree selection in a similar breeding program (same number of fish phenotyped) with a given genotyping tool (9,195 and 11,239 SNP markers obtained by RAD-sequencing for sea bass and sea bream, respectively).

From an economic perspective, the efficiency of a breeding program to improve disease resistance is limited by two main factors. The first one is the cost of the phenotypes. In disease resistance breeding programs, the phenotypes must be recorded on individuals that are closely related to the selection candidates (full and half-sibs) and only produced for this phenotyping purpose. Although natural challenge on the field could be used (Fraslin et al., 2019), the disease challenge is preferred to take place in a dedicated facility to control the infection process (pathogen strain, concentration, and route of infection), to record daily the mortality and to avoid the risk of spreading the pathogen in the wild and to commercial lines (Fjalestad et al., 1993). All those factors make disease resistance phenotyping expensive. The second limiting factor is the necessary genotyping of both selection candidates and challenged relatives. In breeding programs that use pedigree information with communal rearing of families, both selection candidates and challenged individuals are genotyped for a dozen of microsatellite markers or a few hundreds of SNP markers for parentage assignment (Vandeputte and Haffray, 2014). When using genomic selection, they should be genotyped for more SNP markers, typically several thousand or tens of thousands. Even though genotyping becomes more and more affordable, it remains expensive for the majority of breeding companies (Ødegård et al., 2011).

To limit the cost of a disease resistance breeding program, two options are available. The first one is to reduce the number of phenotypes recorded, which results in reduced costs for both genotyping and phenotyping. The second one to reduce the number of markers genotyped, which only decreases the cost of genotyping. Limiting the number of phenotyped individuals, that constitute the training population, affects the accuracy of genomic prediction (Dufflocq et al., 2019). In general, as the number of individuals in the training population increases, the accuracy increases until it reaches a plateau. The density of markers has similar effects, as prediction accuracy increases with the number of markers, until it also reaches a plateau (Tsai et al., 2016; Kriaridou et al., 2020).

There are many other factors that can impact the prediction accuracy, including trait heritability, effective population size (*N_e*), the degree of relatedness between the training population and the validation population, the genetic architecture of the trait (Daetwyler et al., 2010), and the extent of linkage disequilibrium (LD; Vallejo et al., 2018).

The aim of this study was to assess the impact of training population size as well as marker density on the accuracy of genomic selection, compared to a pedigree-based selection, to improve disease resistance in fish. To do so, we used one data set from gilthead sea bream challenged to *P. damselae* subsp. *piscicida*, one data set from European sea bass challenged to *V. harveyi* and two data sets from European sea bass challenged to NNV.

## Materials and Methods

### Ethical Approval

All infection challenges were carried out in accordance with the European guidelines (Directive 2010–63-EU) and the corresponding French legislation. Animal experiment procedures were approved by the ethics committee COMETH n°16 (ANSES, ENVA, and UPEC) and authorized under numbers 14/03/17-10 (n° APAFiS: 2017022816255366), 29/01/13-05 and 10/03/15-01 by the French Ministry of Higher Education, Research and Innovation.

### Fish Material

The animals challenged came from four commercial cohorts from the breeding programs of two different companies. The commercial cohorts were produced by artificial mating and are identified as VNN_A and VNN_B for the two European sea bass cohorts challenged to NNV, VIB for the European sea bass cohort challenged to *V. harveyi* and PAS for the gilthead sea bream cohort challenged to *P. damselae* subsp. *piscicida*. Cohort VNN_A (1,680 individuals) was produced by mating 59 sires with 20 dams in four partial factorial designs (15 × 5, 15 × 5, 15 × 5, and 14 × 5). Cohort VNN_B (1,737 individuals) was generated from 39 sires and 14 dams mated in six factorial subsets (6 × 3, 6 × 1, 6 × 3, 7 × 2, 7 × 3, and 7 × 2). Cohort VIB (2,100 individuals) was produced by mating 60 sires with 18 dams in three factorial subsets (20 × 6, 20 × 7, and 20 × 5) and cohort PAS (1,200 individuals) was produced by mating 50 sires with 23 dams in 6 factorial subsets (8 × 4, 10 × 3, 7 × 4, 10 × 4, 7 × 5, and 8 × 3). The cohorts VNN_A and VNN_B were referred as popA and popB in Griot et al. (2021).

### Infection Challenges

All infection challenges were performed at the SYSAAF-ANSES Fortior Genetics platform (ANSES, Plouzané, France). All fish were individually tagged with RFID glass tags. In each infection challenges, pre-tests were made using 180, 150, 430, and 89 randomly sampled individuals from the VNN_A, VNN_B, VIB, and PAS cohorts, respectively to define the conditions to be used for the challenges.

For the challenge tests themselves, 1,350 individuals from VNN_A (mean body weight = 25 g) and 1,212 individuals from VNN_B (mean body weight = 20 g) were challenged to NNV in filtered and UV sterilized seawater at 27°C ± 2. The infection was done by immersing them in static seawater containing 1 × 10^5^ Tissue Culture Infectious Dose (TCID_50_)/mL of the W80 strain of redspotted grouper nervous necrosis virus (RGNNV; Thiéry et al., 2004) produced on SSN-1 (snakehead fish) cell line for 2 h and 15 min. Then, mortality was recorded daily for 27 days for VNN_A and 42 days for VNN_B.

In the VIB cohort, 1,475 individuals (mean body weight = 15 g) were challenged to *V. harveyi* at 22°C ± 2. The fish were infected by intraperitoneal (IP) injection of 100 μL of the strain 94473 1811603 at a concentration of 2 × 10^8^ Colony Forming Unit (CFU)/fish. The mortality was recorded daily for 13 days.

In the PAS cohort, 960 individuals (mean body weight = 3 g) were challenged to *P. damselae* subsp. *piscicida* at 24°C ± 2. They were infected by IP injection of 100 μL of the strain PP11787 6/94 at a concentration of 3 × 10^11^ CFU/fish. The mortality was recorded daily for 10 days.

For each challenge, we included negative control groups which were IP injected with sterile soy trypticase medium for VIB and PAS cohorts, and immersed in sterile cell culture medium for VNN_A and VNN_B cohorts.

Purity of the bacterial inoculates was controlled after a step of culture on soy trypticase agar by Maldi Tof analysis. The VNN inoculate was controlled using immunofluorescence antibody test (IFAT) after a step of cell culture on SSN-1.

Virological and bacteriological analysis were performed on fish sampled from the quarantine time, on random samples of 10, 4, 5, and 20 individuals taken at the mortality peak from VNN_A, VNN_B, VIB, and PAS cohorts, respectively, and on negative control fish. For VNN, brain, eyes, heart, spleen, and kidney were analyzed by cell culture followed by an IFAT. For *V. harveyi* and *P. damselae* subsp. *piscicida*, bacterial isolation was done from spleens and kidneys and colonies identified using Maldi-Tof.

Binary survival status at the end of the challenge was the phenotype analyzed.

The details of the infection challenge applied to each cohort is summarized in Table 1.

**TABLE 1:** Summary of infection challenges procedure followed by the four commercial cohorts (VNN_A, VNN_B, VIB, and PAS).

	VNN_A	VNN_B	VIB	PAS
Number of individuals	1,680	1,737	2,100	1,200
Number of parents (sires/dams)	59/20	39/14	60/18	50/23
Number of fullsib families	248	69	333	126
Number of halfsib families	79	53	78	73
Number of offspring per fullsib family min–max (mean)	1–21 (5)	1–82 (16)	1–14 (4)	1–44 (8)
Number of individuals in pre-test	180	150	430	89
Number of individuals challenged	1,350	1,212	1,475	960
Pathogen	RGNNV	RGNNV	Vibrio harveyi	Photobacterium damselae subsp. piscicida
Strain	W80	W80	94473 1811603 AQN553P2	PP11787 6/94
Infection method	Immersion	Immersion	IP injection	IP injection
Concentration	1 × 10^5^ TCID/mL	1 × 10^5^ TCID/mL	2 × 10^8^ CFU/fish	3 × 10^11^ CFU/fish
Water temperature (°C)	27 ± 2	27 ± 2	22 ± 2	24 ± 2
Duration of the challenge (in days)	27	42	13	10
Average survival rate	45.2%	59.7%	59.0%	40.0%

*TCID, tissue culture infectious dose; CFU, colony forming unit.*

### Genotyping, Quality Control and Parentage Assignment

Genotyping was performed at the Gentyane genotyping platform (INRAE, Clermont-Ferrand, France). From the challenged individuals and their parents, 1,152 individuals from VNN_A, VNN_B, and 1,151 individuals from VIB were genotyped on the ThermoFisher Axiom^TM^ 57k SNP DlabChip. In the PAS cohort, 1,026 individuals were genotyped on the ThermoFisher Axiom^TM^ 60k SNP SaurChip. Genotyped individuals were sub-sampled from the challenged ones ensuring each sub-sample had the same average survival rate as the whole challenge batch. SNP calling was performed using the ThermoFisher AxiomAnalysisSuite software and quality controls with PLINK 1.9 (Purcell and Chang, 2015). First, individuals with a genotyping rate lower than 90% were discarded. Then, for the sea bass data sets (VNN_A, VNN_B, and VIB), genotyping quality controls were performed on a global population composed of the three data sets. A common set of markers was subset by keeping only markers with a call rate higher than 95%, a minor allele frequency higher than 0.05 and a p-value for the departure from Hardy-Weinberg equilibrium test higher than 10^–8^, resulting in 44,772 common markers to be used for the three cohorts. For the PAS data set, markers with a call rate higher than 95%, a minor allele frequency higher than 0.05 and a p-value for the departure from Hardy-Weinberg equilibrium test higher than 10^–4^ were retained, leaving 43,618 usable markers. When some individuals had missing genotypes for some markers, those missing marker genotypes were imputed with FImpute (Sargolzaei et al., 2014).

Parentage assignment was done using 1,000 randomly sampled markers, analysed with the R package APIS (Griot et al., 2020) with a positive assignment error rate set to 1%.

### Heritability Estimation

For each data set, we estimated the heritability of disease resistance with either a threshold model using THRGIBBSF90 (Tsuruta and Misztal, 2006) or a linear model using AIREMLF90 (Misztal et al., 2002). Only individuals with a phenotype, a genotype and a pedigree were used in heritability estimates, thus the sample size was 1,027 in VNN_A and, 1,042 in VNN_B, 1,049 in VIB, and 916 in PAS.

The following model was computed in each data set using both threshold and linear models:


y=1b+Zu+e

With ***y*** the vector of the phenotypes measured as binary dead/survival trait (0 for dead and 1 for survived), 1 the incidence (unity) vector of the intercept, *b* the estimate of the intercept effect, ***u*** the vector of breeding values and **Z** the corresponding incidence matrix. To compare pedigree-based and genomic-based heritability estimation, *u* followed either a multivariate normal distribution N(0, **A**σ^2^_*g*_) with **A** the pedigree-based relationship matrix or a multivariate normal distribution N(0, **G**2*_*g*_*) with **G** the genomic relationship matrix proposed by VanRaden (2008). σ2_*g*_ is the additive genetic variance and ***e*** is the vector of the random residual errors that follows a normal distribution N(0, ***I***σ^2^_*e*_) with σ^2^_*e*_ the residual variance and ***I*** the identity matrix.

With the threshold model, the variance components (σ^2^_*g*_ and σ^2^_*e*_) were estimated using a Gibbs sampler with 500,000 iterations, 100,000 of burn-in and one sample was kept every 20 iterations for posterior analysis. The residual variance σ^2^_*e*_ was set to a value of 1. The posterior distributions were analyzed with the R package boa to check for correct burn-in size, convergence, no correlation between the sampled iterations and to estimate the variance components (Smith, 2007). With the linear model, the same components were estimated using a restricted maximum likelihood algorithm, considering the observed binary phenotype as a continuous variable.

The heritability for survival was estimated as:


h2=σ2gσ2g+σ2e

Heritability on the observed scale (*h^2^_*o*_*) was estimated using the variance components from the linear model, while the heritability on the underlying liability scale (*h^2^_*u*_*) was computed using the variance components from the threshold model.

### Creation of Virtual Low-Density SNP Chips

From the SNP markers obtained after quality controls, four low-density (1K, 3K, 6K, and 10K) virtual SNP chips were created. To do so, we used a marker pruning method based on the LD (Porto-Neto et al., 2013). In a user-defined sliding window, every pairwise LD between markers was estimated by the r^2^ metrics. Then, SNPs were pruned until no pair had a r^2^ greater than a given value, until we reached the desired number of SNPs.

For the creation of the sea bass 10K chip, we used an iterative method with a sliding window of 1 Mb and a r^2^ value of 0.434. First, the SNP were pruned in the cohort VNN_A data set with the LD method explained above. Then, the remaining markers in cohort VNN_A were subset from cohort VNN_B and the same pruning method was applied on the remaining ones. The same process was done one last time on the cohort VIB data set to obtain the desired number of markers. Finally, the remaining markers in the VIB data set were subset in cohort VNN_A and cohort VNN_B data sets to obtain 10,020 markers in each cohort.

The same process was repeated to create the 1K, 3K, and 6K chips by modifying the sliding window size of 100, 200, and 500 kb and the r^2^ threshold value of 0.175, 0.268, and 0.344 for 1K, 3K, and, 6K, respectively. The virtual chips for sea bass contained 1,007, 3,022, 6,010, and 10,020 markers for the 1K, 3K, 6K, and 10K, respectively.

For the PAS data set, the pruning was done by applying the same protocol, but on one dataset only. The chips contained 1,007, 2,999, 6,011, and 10,010 markers for the 1K, 3K, 6K, and 10K, respectively and were done by using a sliding window of 100, 200, 500, 1,000 kb, and a r^2^ threshold of 0.044, 0.191, 0.318, and 0.434 for the 1K, 3K, 6K, and 10K, respectively.

In each species, the size of the sliding window as well as the r^2^ value for LD were empirically chosen to uniformly sample the desired number of markers.

### Creation of the Training Population

We fixed the validation population size to 200 individuals randomly chosen from the whole population in each data set. The training population was composed of a minimum of 50 individuals up to 800 in sea bass data sets and 700 in sea bream data set. From 50 to 200, we added 50 individuals at each step and then, from 200, we added 100 individuals per step. The initial 50 individuals were randomly sampled from remaining individuals after the validation population had been chosen. Then, to increase the training population size, the added individuals were randomly chosen from the remaining individuals and added to the previous training population. This process was repeated until the training validation population size reached the maximum limit. In all sea bass data sets (VNN_A, VNN_B, and VIB), we tested training population sizes of 50, 100, 150, 200, 300, 400, 500, 600, 700, and 800 individuals. In the PAS cohort, we tested training population sizes of 50, 100, 150, 200, 300, 400, 500, 600, and 700 individuals. To account for stochastic sampling effects, the entire process was repeated 100 times for each data set. This approach is similar to the “Monte-Carlo cross-validation” proposed by Kuhn and Johnson (2013) and applied in D’Ambrosio et al. (2020).

### Effect of the Density of Markers and Training Population on Prediction Accuracy

For each replicate of training population size, we tested six densities of markers. We tested the low-density SNP chips (1K, 3K, 6K, and 10K) as well as the full SNP chip (44K for sea bass data sets and 43K for sea bream data set) and a control case with only pedigree information and no genomic information.

The phenotypes of the individuals in the validation population were masked and the breeding values were estimated with the same linear model as the one described in section “Heritability Estimation,” adjusted with AIREMLF90, using the genomic relationship matrix when genomic information was used (GBLUP) or the pedigree-based relationship matrix when only pedigree information was used (PBLUP). The accuracy was computed as:


$$r=\frac{cor\left(EBV,y\right)}{h}$$

With *cor*(*EBV*, *y*) the correlation between the EBV and the phenotypes y of the 200 individuals belonging to the validation population and h the square-root of the heritability estimated with a linear model, using the pedigree-based relationship matrix and the whole data set as in the section “Heritability Estimation.” The accuracies of the 100 replicates for each training population size and marker density were averaged.

### Addition of the QTL Effect on Prediction Accuracy in VNN Resistance

In Griot et al. (2021), the authors showed that one strong effect QTL is involved in VNN resistance in European sea bass. Among the markers within the confidence interval, they highlighted one marker (LG12_8815613) with high potential for marker assisted selection. Here, we assessed the impact of adding the QTL effect in the estimation of breeding values on the prediction accuracy. To do so, in the cohorts VNN_A and VNN_B, the model described in section “Effect of the density of markers and training population on prediction accuracy” was replaced by the following model as proposed by Kennedy et al. (1992):


y=Xb+Zu+e

With all parameters remaining the same except for **X***b* where **X** is the genotype matrix for the LG12_8815613 marker coded as 0 for the homozygous, 1 for the heterozygous and 2 for the alternative homozygous and b is the marker fixed effect.

Then, the GEBV were computed as:


$$GEBV=\text{û}+\text{X}\hat{b}$$

With û the estimated additive effect of the polygenic breeding value and $\hat{b}$ the estimated QTL effect.

Finally, accuracy was estimated as in section “Effect of the Density of Markers and Training Population on Prediction Accuracy” only for a training population of 800 individuals.

### Estimation of Linkage Disequilibrium

The LDbetween each pair of SNPs was estimated using the Pearson correlation (r^2^). Pairwise LD within each chromosome for each cohort was estimated with the software PREGSF90 from the blupf90 program suite. LD extent estimation was performed using the same approach as proposed in Barría et al. (2019). All pairs of SNPs were sorted based on the distance between the two markers. Then, the r^2^ was averaged over all markers within a bin of 100 kb, up to a distance of 20 Mb (0–99 kb, 100–199 kb, …).

## Results

### Challenges

The four challenges were conducted up to 42 days. The presence of the different pathogen agents in their respective challenges was confirmed by virologic or bacteriological analyses on random samples of fish that died during the challenges. All control fish were negative to the pathogen. In VNN_A and VNN_B cohorts’ challenges, fish showed clear clinical sign of the disease and NNV was detected in all analysed fish (*n* = 10 for VNN_A et *n* = 4 for VNN_B). Similarly, *V. harveyi* was detected in 100% of the fish samples (*n* = 5) from the VIB cohort challenge. In PAS cohorts challenge, only four fish over 20 were positive to *P. damselae* subsp. *piscicida*. Different species of *vibrio* bacteria (*Aliivibrio fischeri*, *vibrio rotiferianus*, and *V. harveyi*) were punctually detected.

**FIGURE 1:**
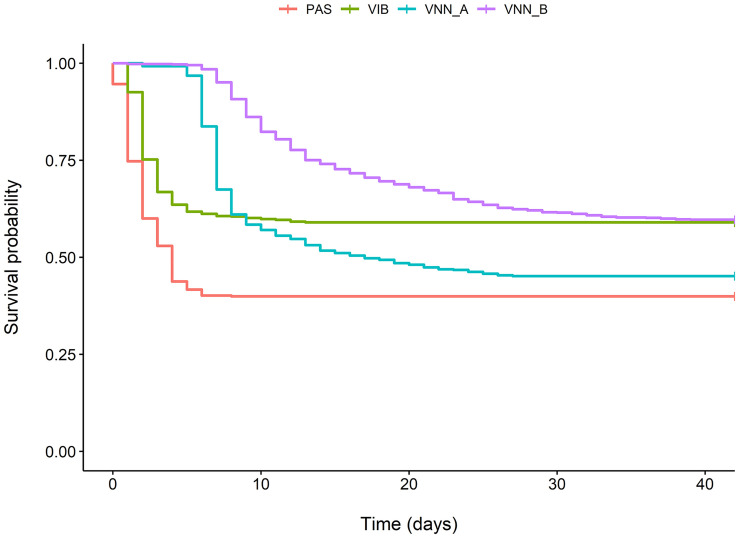
Kaplan-Meier probability of survival over time following infection for two European sea bass commercial cohorts challenged to NNV (VNN_A and VNN_B), one European sea bass commercial cohort challenged to *V. harveyi* (VIB) and one gilthead sea bream commercial cohort challenged to *Photobacterium damselae* subsp. *piscicida* (PAS).

Survival rates ranging from 40.0 to 59.7% were recorded (Figure 1). The mortality peaks were early for challenges to *V. harveyi* and *P. damselae* subsp. *piscicida*, at 3 and 2 days, respectively. For NNV, the peak was at 9 days post infection, and was much sharper in cohort VNN_A than in cohort VNN_B.

### Heritability Estimation

Heritability was moderate for all diseases, ranging from 0.103 to 0.238 using a pedigree-based linear model and from 0.111 to 0.232 using a genomic linear model (Table 2). For all challenges, genomic and pedigree based heritability estimates were very similar. When using the threshold model, the estimates were much higher than those estimated with the linear models, ranging from 0.198 to 0.421 for pedigree-based heritability and from 0.198 to 0.379 for genomic heritability.

**TABLE 2:** Heritability estimated for Viral Nervous Necrosis (VNN) resistance in two European sea bass commercial cohorts (VNN_A and VNN_B), vibriosis resistance in one European sea bass commercial cohort (VIB) and pasteurellosis resistance in one gilthead sea bream commercial cohort (PAS) with pedigree-BLUP (PBLUP) or genomic-BLUP (GBLUP) using linear or threshold models using full density chips.

Population	PBLUP		GBLUP	
	Linear model	Threshold model	Linear model	Threshold model
VNN_A	0.238 (±0.063)	0.421 (±0.106)	0.232 (±0.049)	0.379 (±0.065)
VNN_B	0.103 (±0.048)	0.214 (±0.087)	0.118 (±0.043)	0.217 (±0.068)
VIB	0.109 (±0.043)	0.198 (±0.068)	0.111 (±0.040)	0.198 (±0.064)
PAS	0.139 (±0.051)	0.291 (±0.086)	0.159 (±0.045)	0.295 (±0.066)

*Values in parenthesis are the standard errors.*

### Effect of Training Population Size and Marker Density on Prediction Accuracy

**FIGURE 2:**
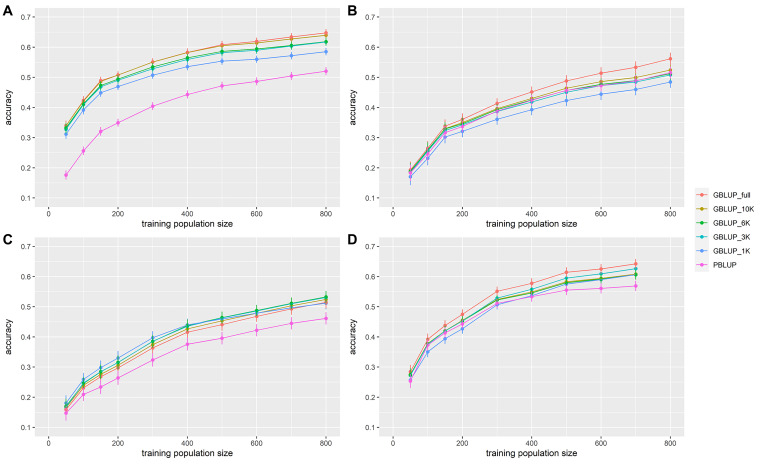
Accuracy of genomic (GBLUP) and pedigree-based (PBLUP) estimated breeding values for disease resistance as a function of the number of individuals in the training population, and for different marker densities, in **(A)** European sea bass commercial cohort VNN_A challenged to NNV, **(B)** European sea bass commercial cohort VNN_B challenged to NNV, **(C)** European sea bass commercial cohort VIB challenged to *V. harveyi*, and **(D)** gilthead sea bream commercial cohort PAS challenged to *Photobacterium damselae* subsp. *piscicida*. Each point is the average of 100 replicates. Error bars represent the standard error of the mean of 100 replicates.

In all data sets, accuracy increased with the size of the training population (Figure 2 and Table 3). From 50 to 150 individuals in the training population, the increase in accuracy was the greatest. From 150 to 400–500 individuals in the training population, the increase in prediction accuracy was intermediate and with more than 400–500 individuals in the training population, the prediction accuracy increased slowly. With the full density chip, GBLUP with a training population size of 200, 500, 500, and 300 individuals reached the same accuracy as PBLUP for VNN_A, VNN_B, VIB, and PAS, respectively.

**TABLE 3:** Prediction accuracy for VNN resistance in two European sea bass commercial cohorts (VNN_A and VNN_B), vibriosis resistance in one European sea bass commercial cohort (VIB) and pasteurellosis resistance in one gilthead sea bream commercial cohort (PAS) using different training population sizes and marker densities.

Data set	Training population size	PBLUP	GBLUP_1K	GBLUP_3K	GBLUP_6K	GBLUP_10K	GBLUP_full
10*VNN_A	50	0.18	0.31	0.33	0.34	0.33	0.34
	100	0.26	0.39	0.41	0.42	0.41	0.42
	150	0.32	0.45	0.47	0.49	0.47	0.49
	200	0.35	0.47	0.49	0.51	0.49	0.51
	300	0.40	0.51	0.53	0.55	0.53	0.55
	400	0.44	0.54	0.56	0.58	0.56	0.58
	500	0.47	0.55	0.58	0.61	0.59	0.61
	600	0.49	0.56	0.59	0.62	0.59	0.61
	700	0.50	0.57	0.60	0.63	0.61	0.63
	800	0.52	0.59	0.62	0.65	0.62	0.64
10*VNN_B	50	0.18	0.17	0.18	0.19	0.19	0.19
	100	0.25	0.23	0.26	0.26	0.26	0.26
	150	0.32	0.30	0.32	0.34	0.33	0.33
	200	0.34	0.32	0.34	0.36	0.35	0.35
	300	0.39	0.36	0.39	0.41	0.39	0.40
	400	0.42	0.39	0.42	0.45	0.43	0.43
	500	0.46	0.42	0.45	0.49	0.46	0.46
	600	0.47	0.44	0.47	0.51	0.48	0.49
	700	0.49	0.46	0.48	0.53	0.49	0.50
	800	0.52	0.48	0.51	0.56	0.51	0.52
10*VIB	50	0.15	0.18	0.17	0.16	0.17	0.17
	100	0.21	0.26	0.25	0.23	0.25	0.24
	150	0.23	0.30	0.28	0.27	0.28	0.28
	200	0.26	0.33	0.31	0.30	0.32	0.31
	300	0.32	0.40	0.39	0.36	0.39	0.37
	400	0.38	0.44	0.44	0.42	0.44	0.43
	500	0.40	0.46	0.46	0.44	0.46	0.45
	600	0.42	0.48	0.49	0.47	0.49	0.48
	700	0.45	0.50	0.51	0.49	0.51	0.50
	800	0.46	0.51	0.53	0.51	0.53	0.52
9*PAS	50	0.25	0.26	0.27	0.28	0.27	0.28
	100	0.37	0.35	0.38	0.39	0.38	0.38
	150	0.41	0.39	0.42	0.44	0.42	0.42
	200	0.44	0.43	0.45	0.47	0.45	0.46
	300	0.51	0.51	0.53	0.55	0.52	0.52
	400	0.53	0.54	0.56	0.58	0.55	0.55
	500	0.56	0.58	0.59	0.61	0.58	0.58
	600	0.56	0.59	0.61	0.63	0.59	0.59
	700	0.57	0.61	0.63	0.64	0.61	0.61

*Prediction accuracy values are averaged over 100 replicates.*

**FIGURE 3:**
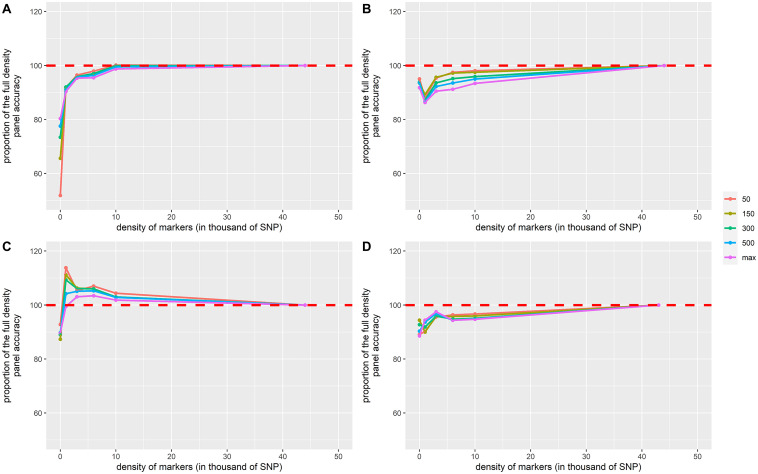
Proportion of the full-density SNP panel accuracy for genomic breeding value estimates as a function of the density of markers in **(A)** European sea bass commercial cohort VNN_A challenged to NNV, **(B)** European sea bass commercial cohort VNN_B challenged to NNV, **(C)** European sea bass commercial cohort VIB challenged to *V. harveyi*, and **(D)** gilthead sea bream commercial cohort PAS challenged to *Photobacterium damselae* subsp. *piscicida*. The density of markers is expressed in thousands of SNPs. Only training population size of 50, 150, 300, 500, and 700 for PAS and 800 for others are displayed and represented by the color palette. Each point is the average of 100 replicates.

In general, accuracy increased with the density of markers (Figure 2 and Table 3). The addition of genomic information improved the accuracy compared to that of pedigree-based estimation, except in VNN_B where the accuracy estimated with PBLUP was greater than that estimated with 1,000 markers (Figure 2B). With the maximum training population and the full density chip, genomic evaluation led to an increase in accuracy, compared to PBLUP, of 24.5, 8.9, 11.6, and 12.9% for VNN_A, VNN_B, VIB, and PAS, respectively. Except in VNN_B, the use of one thousand markers for genomic evaluation increased the accuracy by 12.5, 11.0, and 6.6% for VNN_A, VIB, and PAS compared to PBLUP. In general, a density of 6K was enough to reach at least 90% of the accuracy obtained with the full density chip (Figure 3 and Table 4).

**TABLE 4:** Relative prediction accuracy of estimated breeding values (EBV) (in %) compared to GBLUP_full for Viral Nervous Necrosis (VNN) resistance in two European sea bass commercial cohorts (VNN_A and VNN_B), vibriosis resistance in one European sea bass commercial cohort (VIB) and pasteurellosis resistance in one gilthead sea bream commercial cohort (PAS) using different training population sizes and marker densities.

Data set	Training population size	PBLUP	GBLUP_1K	GBLUP_3K	GBLUP_6K	GBLUP_10K
VNN_A	50	51.8	91.7	96.5	97.9	100.1
	150	65.6	91.8	95.8	96.7	99.5
	300	73.4	92.0	95.9	97.0	99.9
	500	77.6	91.0	95.7	96.4	99.5
	800	80.3	90.4	95.3	95.5	98.7
VNN_B	50	95.0	88.1	95.3	97.5	98.0
	150	93.5	89.2	95.6	97.2	97.5
	300	93.8	87.3	93.6	95.1	95.9
	500	93.6	86.6	92.2	93.5	95.0
	800	91.8	86.3	90.5	91.2	93.4
VIB	50	92.8	113.8	105.3	107.0	104.4
	150	87.3	111.2	106.3	106.0	103.0
	300	89.0	109.4	106.1	106.0	103.0
	500	89.7	104.2	105.1	105.3	102.8
	800	89.6	99.5	103.0	103.4	101.8
PAS	50	89.1	90.2	95.6	96.4	96.7
	150	94.4	90.0	95.8	95.8	96.0
	300	92.7	91.9	95.9	94.8	95.1
	500	90.3	93.6	96.8	94.3	94.9
	700	88.6	94.4	97.5	94.3	94.7

### Addition of the QTL Effect on Prediction Accuracy in VNN Resistance

**FIGURE 4:**
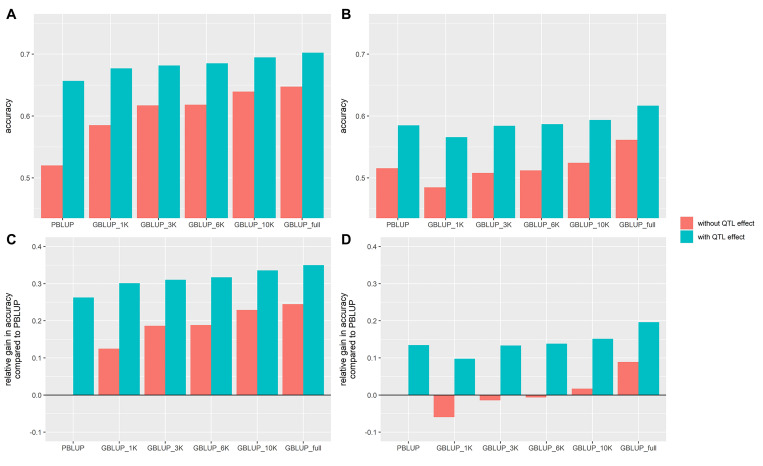
Accuracy of genomic (GBLUP) and pedigree-based (PBLUP) estimated breeding values for VNN resistance in two European seabass commercial cohorts (VNN_A, **A** and VNN_B, **B**) with different SNP chip densitiesand with (in blue) or without (in red) the QTL effect and a training population of 800 individuals. Relative gain in accuracy compared to the GBLUP_full model ignoring the QTL effect in cohort VNN_A **(C)** and VNN_B **(D)**.

The addition of the QTL effect increased the prediction accuracy in a range of 10.5–26.3% compared to the prediction accuracy estimated without it (Figure 4). In every cohort and for every chip density, it led to an increase in prediction accuracy. The prediction accuracy using just the pedigree and the QTL information was slightly higher than that of the full density chip GBLUP for either VNN_A or VNN_B (Figures 4C,D).

### Estimation of Linkage Disequilibrium

**FIGURE 5:**
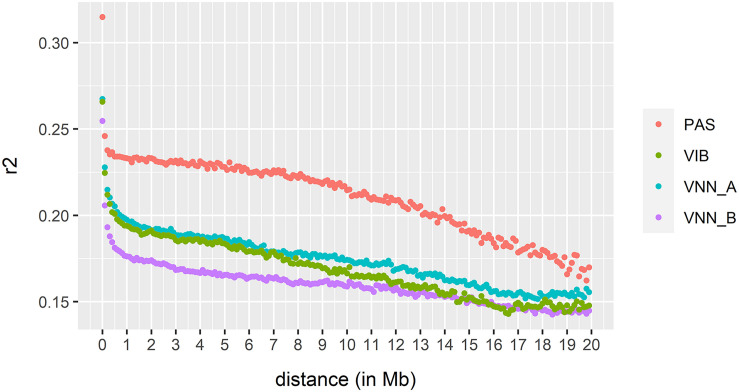
Extent of linkage disequilibrium estimated in two European sea bass commercial cohorts VNN_A and VNN_V challenged to NNV, one European sea bass commercial cohort VIB challenged to *V. harveyi* and one gilthead sea bream commercial cohort PAS challenged to *Photobacterium damselae* subsp. *piscicida*.

As expected, LD decreased rapidly up to 0.5 Mb in all cohorts (Figure 5). The average LD estimates were 0.174, 0.160, 0.169, and 0.210 for VNN_A, VNN_B, VIB, and PAS cohort, respectively. In general, the LD of all cohorts decreased at the same rate up to a distance of 10 Mb. Then, LD decreased faster in the PAS (sea bream) cohort compared to all sea bass cohorts.

## Discussion

Genomic selection is widely recognized as having a great potential to improve selective breeding (Meuwissen et al., 2016). In the context of improving pathogen resistance in aquaculture species, its relevance has already been shown (Vallejo et al., 2017; Palaiokostas et al., 2018, 2016). In the present work, we provided essential data to implement genomic selection to improve disease resistance to the most common pathogens in European sea bass and gilthead sea bream aquaculture. First, we obtained moderate heritability estimates for resistance to the three pathogens. With the threshold model, the pedigree-based and genomic heritability estimates were rather similar, except for VNN_A for which the pedigree-based heritability estimate was higher than the genomic heritability estimate. The use of genomic information mainly reduced the standard error of the estimates and thus, improved their precision. The heritability estimates were very different between linear model and threshold model, even after applying the correction of the linear estimates proposed by Dempster and Lerner (1950) (data not shown). Our heritability estimates were similar to those reported in former studies, when available: for VNN resistance in sea bass, threshold model heritability estimates were in the range 0.21–0.42, to be compared to previous values of 0.24–0.43 estimated using threshold model or linear model corrected using Dempster and Lerner formula (Doan et al., 2017; Palaiokostas et al., 2018; Griot et al., 2021). For pasteurellosis resistance in sea bream, our estimates from linear model (0.14–0.16) were in the lower range of those from previous studies, estimated using linear model (0.22–0.45) (Antonello et al., 2009; Palaiokostas et al., 2016; Aslam et al., 2018). For vibriosis resistance in sea bass, we presented the first genetic parameters estimates to our knowledge. In other aquaculture species, vibriosis resistance has shown low to moderate heritability, ranging from 0.13 in Atlantic salmon on *Vibrio salmonicida* to 0.19 in Chinese tongue sole on *V. harveyi* using a linear model and 0.16 in Atlantic cod on *Vibrio anguillarum* using a threshold sire-dam model (Gjedrem and Gjøen, 1995; Bangera et al., 2011; Li et al., 2019). Similar results were found on shellfish, with moderate heritability of 0.11 in white shrimp on *Vibrio parahaemolyticus* and 0.09–0.33 in Pacific oysters on *Vibrio aestuarianus* (Azéma et al., 2017; Lyu et al., 2020). Heritability estimates for all studied diseases were moderate, meaning that resistance to all these diseases can be improved by selective breeding.

We showed that genomic selection using a 44K SNP chip can improve the accuracy of breeding values in the range 9–25% compared to pedigree-based selection. Palaiokostas et al. (2018) showed an increase of 8% of the prediction accuracy using a GBLUP model with 9,195 markers compared to a PBLUP model for VNN resistance in sea bass. In this paper, they used the area under curve (AUC) as a metric for measuring accuracy. As our measure is very different, we cannot compare the results. However, we both showed that genomic selection outperformed pedigree-based selection to improve VNN resistance in sea bass. In Palaiokostas et al. (2016), the authors reached a prediction accuracy of 0.44 using a GBLUP model compared to 0.30 when using a PBLUP model for pasteurellosis resistance in gilthead seabream using 578 individuals in the training population and 200 individuals in the validation population. Here, even though the improvement of prediction accuracy using GBLUP was lower than in that previous study [+43% in Palaiokostas et al. (2016) versus +13% in our work], we obtained much higher absolute values (0.56 using PBLUP and 0.63 using GBLUP with 600 individuals in the training population). Such a difference could be explained by different mating design or genetic diversity within each population.

We showed that an increase in training population size led to an increase in prediction accuracy. Accuracy changed with training population size in a specific way in each data set. Population structure and heritability are two major drivers for the change in accuracy as function of the training population size (Goddard, 2009; Meuwissen et al., 2013). Even though cohorts VNN_A and VIB came from similar mating designs and were genotyped with the same markers, they had very different accuracy profiles (Figures 2A,C). The absolute values of accuracy were different and the increase in accuracy between 50 and 150 individuals in the training population was greater in VNN_A than in VIB. The heritability was very different between the two traits, as well as the average survival rate, and both have impact on the accuracy. One other factor that can explain the difference in accuracy between traits is the genetic architecture. As VNN resistance was reported to be an oligogenic trait (Griot et al., 2021), a model that can take advantage of genetic architectures that are not polygenic (wssGBLUP or BayesB) could perform better than a GBLUP model (Daetwyler et al., 2010; Vallejo et al., 2017).

Across all the data sets, even though accuracy increased less and less as the training population size increased, we did not reach the plateau. In fact, in most of the aquaculture breeding programs, prediction accuracy plateau is reached when the training population size is 4,000 individuals or more (Lillehammer et al., 2013; Dagnachew and Meuwissen, 2019), but this could not be tested here due to the limitation in the number of phenotyped and genotyped fish for each disease. Thus, adding more individuals from the same generation or of successive generations would still increase prediction accuracy (Figure 2). Another way to increase prediction accuracy at a constant training population size could be an optimized choice of the training population. By doing this using an optimization algorithm, the prediction accuracy could be significantly improved compared to a random selection of the training population (Rincent et al., 2012; Akdemir et al., 2015). However, the absolute size of the training population is the main driver compared to its composition (Bradford et al., 2017).

Across all diseases and species we studied, between 3K and 6K SNP markers were enough to obtain a high prediction accuracy. Similar results were found in other aquaculture species (Kriaridou et al., 2020). In Kriaridou et al. (2020), the authors found that a marker density between 1K and 2K was sufficient to keep the prediction accuracy close to that of the full density chip. In our study, such a density would lead to a significant decrease in prediction accuracy. An explanation could be that we compared the prediction accuracy of low-density SNP panels to a 43K or 44K SNP chip, while Kriaridou et al. (2020) compared the low-density SNP panels to a 10K SNP chip. If we compared the prediction accuracy of the 1K, 3K, and 6K SNP chips to the 10K, the 3K chip maintained the accuracy to 95% of the value of the 10K and the 1K chip to 90% of the value of the 10K, which is close to the values obtained in the study of Kriaridou et al. (2020). In rainbow trout, a prediction accuracy greater than the one obtained with PBLUP can be as achieved with only 500 (Vallejo et al., 2018). Such high accuracy with low number of markers was explained by the extent of long-range LD within the species.

Here, the extent of long-range LD is lower than that observed in rainbow trout, which could explain the lower difference between PBLUP and GBLUP_1K. The level of LD over 1 Mb was greater than 0.25 in rainbow trout (Vallejo et al., 2018), but much lower in our cohorts (*r*^2^ = 0.2, *r*^2^ = 0.18, *r*^2^ = 0.19, and *r*^2^ = 0.23 in VNN_A, VNN_B, VIB, and PAS cohort). However, we cannot see any clear evidence of an effect of LD on the prediction accuracy, as accuracy is not clearly correlated with LD in our 4 cohorts.

Training population size and marker density are two major cost drivers in a genomic selection breeding program (Riedelsheimer and Melchinger, 2013; Rajsic et al., 2016). Both impact the cost of genotyping, while training population size also involves the cost of phenotyping. We showed that a 6K–10K SNP chip was enough to reach at least 90% of accuracy obtained with the full density chip (43–44K). Combining medium to high density genotyping on parents and low density genotyping followed by an imputation on the offspring can be a viable genotyping strategy to reduce the overall cost of the breeding program (Cleveland and Hickey, 2013; Tsai et al., 2017; Tsairidou et al., 2020). Both Tsai et al. (2017) and Tsairidou et al. (2020) showed a significant improvement of the prediction accuracy using imputed SNP data rather than low-density panels. Tsai et al. (2017) showed that the imputation from 256 SNP to 25K increased the prediction accuracy by 45% (from 0.4 to 0.58) for sea lice resistance in Atlantic salmon. Tsairidou et al. (2020) showed that genotyping the parents with a 5K chip and the offspring with only 200 SNP markers then imputing them to the 5K chip led to a prediction accuracy close to the value obtained by the medium density chip, while decreasing the genotyping cost of the breeding program by 62%. With a constant budget for genotyping and infection challenge, a breeder could increase the number of individuals in the training population and, thus, the prediction accuracy or, with the money saved, start other breeding programs on other traits.

In Griot et al. (2021), we showed that one strong effect QTL that explained 9.2% of the genetic variance, was involved in VNN resistance in European sea bass. By adding the information of the marker proposed as marker-assisted selection in the prediction model, we showed a significant increase in the prediction accuracy. The information of the marker genotype in a PBLUP model led to an accuracy slightly higher than that obtained with the 44K chip. In these populations and for this generation, marker-assisted selection would thus seem to be a very relevant choice. However, as mentioned in Griot et al. (2021), this result may not be consistent in other populations and/or generations as this marker was not in complete LD with the QTL. In addition, even though the marker LG12_8815613 had a strong effect in all the populations from Griot et al. (2021), it was selected partly from the same populations in which its effect was tested, this could lead to an overestimation of its effect and thus, a greater accuracy than the one that could be expected in an unrelated population (Phocas, pers. Communication, 2020).

One major driver of prediction accuracy is the degree of relatedness between the training and the validation population (Habier et al., 2007; Pszczola et al., 2012). In aquaculture breeding programs to improve disease resistance traits, the reference population is generally constituted of challenged individuals that are full or half-sibs of the candidates (Ødegård et al., 2011). In that scenario, the degree of relatedness between the training population (composed of the individuals that were challenged) and the candidates is high and thus, high prediction accuracy can be achieved. This is the scenario applied in that study. However, in real life, the goal is to improve disease resistance in the next generation, and this can only be tested through progeny testing (Vallejo et al., 2020). In our case, this was not possible as we currently have only one generation of genomically evaluated fish.

In this study, we presented a framework to implement genomic selection for disease resistance in European sea bass and gilthead sea bream. The results showed that, across all diseases and cohorts, 6,000 SNP markers were sufficient to get high prediction accuracy, equivalent to at least 90% of accuracy reached with the full density chip. For the training population size, as the plateau of accuracy was not reached with 800 individuals, an increase in its size would lead to an increase in accuracy and thus, in genetic gain. For VNN resistance, as one major effect QTL was detected, we showed that marker-assisted selection was an efficient method to improve the prediction accuracy.

## Data Availability Statement

The data that support the findings of this study are available from the breeding companies “Ferme Marine du Douhet” and “Ecloserie Marine de Gravelines-Ichtus” but restrictions apply to the availability of these data, which were used under license for the current study, and so are not publicly available. The data can be made available for reproduction of the results from Jean-Sébastien Bruant (bruant@douhet.fr) or Aline Bajek (aline.bajek@gloriamaris.com) on request via a material transfer agreement and with permission of the breeding companies “Ferme Marine du Douhet” and “Ecloserie Marine de Gravelines-Ichtus”.

## Ethics Statement

The animal study was reviewed and approved by COMETH n°16.

## Author Contributions

FA, PH, and MV designed the project. J-SB, SC, BP, and JB created and reared the fish cohorts. YF and TM performed the infectious challenges. RG performed the analyses. RG, FA, FP, SB-F, RM, PH, AB, and MV analyzed the results. RG wrote the manuscript. FA, FP, PH, and MV reviewed the manuscript. All authors contributed to the article and approved the submitted version.

## Conflict of Interest

RG, SB-F, RM, AB, YF, and PH are employed by SYSAAF, that provides expertise to the management of aquaculture breeding programs in France. SC, JB, J-SB, and BP are employed by companies that run fish breeding programs. The remaining authors declare that the research was conducted in the absence of any commercial or financial relationships that could be construed as a potential conflict of interest.
