# Corrigendum: Optimization of Genomic Selection to Improve Disease Resistance in Two Marine Fishes, The European Sea Bass (*Dicentrarchus labrax*) and the Gilthead Sea Bream (*Sparus aurata*)

**DOI:** 10.3389/fgene.2021.754416

**Published:** 2021-10-11

**Authors:** Ronan Griot, François Allal, Florence Phocas, Sophie Brard-Fudulea, Romain Morvezen, Pierrick Haffray, Yoannah François, Thierry Morin, Anastasia Bestin, Jean-Sébastien Bruant, Sophie Cariou, Bruno Peyrou, Joseph Brunier, Marc Vandeputte

**Affiliations:** ^1^ SYSAAF, Station LPGP/INRAE, Campus de Beaulieu, Rennes, France; ^2^ Université Paris-Saclay, INRAE, AgroParisTech, GABI, Jouy-en-Josas, France; ^3^ MARBEC, Univ. Montpellier, Ifremer, CNRS, IRD, Palavas-les-Flots, France; ^4^ ANSES, Ploufragan-Plouzané-Niort Laboratory, Viral Fish Diseases Unit, National Reference Laboratory for Regulated Fish Diseases, Technopôle Brest-Iroise, Plouzané, France; ^5^ Ferme Marine du Douhet, La Brée Les Bains, France; ^6^ Ecloserie Marine de Gravelines-Ichtus, Gravelines, France

**Keywords:** genomic selection, *dicentrarchus labrax*, *sparus aurata*, disease resistance, aquacultrure

In the original article, there was a mistake in Table 3 as published. Values in column “PBLUP” and “GBLUP_full” are inverted. The corrected Table 3 appears below. The authors apologize for this error and state that this does not change the scientific conclusions of the article in any way. The original article has been updated.

**TABLE 3:** Prediction accuracy for VNN resistance in two European sea bass commercial cohorts (VNN_A and VNN_B), vibriosis resistance in one European sea bass commercial cohort (VIB) and pasteurellosis resistance in one gilthead sea bream commercial cohort (PAS) using different training population sizes and marker densities.

Data set	Training population size	PBLUP	GBLUP_1K	GBLUP_3K	GBLUP_6K	GBLUP_10K	GBLUP_full
VNN_A	50	0.18	0.31	0.33	0.34	0.33	0.34
	100	0.26	0.39	0.41	0.42	0.41	0.42
	150	0.32	0.45	0.47	0.49	0.47	0.49
	200	0.35	0.47	0.49	0.51	0.49	0.51
	300	0.40	0.51	0.53	0.55	0.53	0.55
	400	0.44	0.54	0.56	0.58	0.56	0.58
	500	0.47	0.55	0.58	0.61	0.59	0.61
	600	0.49	0.56	0.59	0.62	0.59	0.61
	700	0.50	0.57	0.60	0.63	0.61	0.63
	800	0.52	0.59	0.62	0.65	0.62	0.64
VNN_B	50	0.18	0.17	0.18	0.19	0.19	0.19
	100	0.25	0.23	0.26	0.26	0.26	0.26
	150	0.32	0.30	0.32	0.34	0.33	0.33
	200	0.34	0.32	0.34	0.36	0.35	0.35
	300	0.39	0.36	0.39	0.41	0.39	0.40
	400	0.42	0.39	0.42	0.45	0.43	0.43
	500	0.46	0.42	0.45	0.49	0.46	0.46
	600	0.47	0.44	0.47	0.51	0.48	0.49
	700	0.49	0.46	0.48	0.53	0.49	0.50
	800	0.52	0.48	0.51	0.56	0.51	0.52
VIB	50	0.15	0.18	0.17	0.16	0.17	0.17
	100	0.21	0.26	0.25	0.23	0.25	0.24
	150	0.23	0.30	0.28	0.27	0.28	0.28
	200	0.26	0.33	0.31	0.30	0.32	0.31
	300	0.32	0.40	0.39	0.36	0.39	0.37
	400	0.38	0.44	0.44	0.42	0.44	0.43
	500	0.40	0.46	0.46	0.44	0.46	0.45
	600	0.42	0.48	0.49	0.47	0.49	0.48
	700	0.45	0.50	0.51	0.49	0.51	0.50
	800	0.46	0.51	0.53	0.51	0.53	0.52
PAS	50	0.25	0.26	0.27	0.28	0.27	0.28
	100	0.37	0.35	0.38	0.39	0.38	0.38
	150	0.41	0.39	0.42	0.44	0.42	0.42
	200	0.44	0.43	0.45	0.47	0.45	0.46
	300	0.51	0.51	0.53	0.55	0.52	0.52
	400	0.53	0.54	0.56	0.58	0.55	0.55
	500	0.56	0.58	0.59	0.61	0.58	0.58
	600	0.56	0.59	0.61	0.63	0.59	0.59
	700	0.57	0.61	0.63	0.64	0.61	0.61

Prediction accuracy values are averaged over 100 replicates.

